# How do cultural elements shape speak-up behavior beyond the patient safety context? An interprofessional perspective in an obstetrics and gynecology department

**DOI:** 10.3389/fmed.2024.1345316

**Published:** 2024-09-04

**Authors:** Romana F. Malik, Poyan Azar, Achraf Taimounti, Martina Buljac-Samardžić, Carina G. J. M. Hilders, Fedde Scheele

**Affiliations:** ^1^Department of Research in Education, OLVG Hospital, Amsterdam, Netherlands; ^2^Athena Institute, Faculty of Science, VU Amsterdam, Amsterdam, Netherlands; ^3^Department of Human Resources, Bunge, Zaandam, Netherlands; ^4^Faculty of Behavioral and Movement Sciences, VU Amsterdam, Amsterdam, Netherlands; ^5^Erasmus School of Health Policy & Management, Erasmus University, Rotterdam, Netherlands; ^6^Reinier de Graaf Hospital, Delft, Netherlands; ^7^Department of Research in Education, Amsterdam University Medical Centre, Amsterdam, Netherlands

**Keywords:** organizational culture, speak-up, interprofessional education, leadership, hierarchy, postgraduate training, healthcare professionals, communication

## Abstract

**Introduction:**

Interprofessional working and learning thrives with speak-up behavior. Efforts to improve speak-up have mainly focused on isolated techniques and training programs within the patient safety scope, yet sustained improvement requires a cultural shift beyond this scope. This research investigates the influence of culture elements on speak-up behavior in interprofessional teams beyond the patient safety context.

**Methods:**

An exploratory qualitative study design was used in a Dutch hospital’s Obstetrics and Gynecology department. A representative sample of stakeholders was purposefully selected, resulting in semi-structured interviews with 13 professionals from different professional backgrounds (nurses, midwifes, managers, medical specialists, and residents). A speak-up pledge was developed by the research team and used to prime participants for discussion. Data analysis involved three-step coding, which led to the development of themes.

**Results:**

This study has identified six primary cultural themes that enhance speak-up behavior. These themes encompass the importance of managing a shared vision, the role of functional hierarchy, the significance of robust interpersonal relationships, the formulation of a strategy delineating when to speak up and when to exercise restraint, the promotion of an open-minded professional mindset, and the integration of cultural practices in the context of interprofessional working and learning.

**Conclusion:**

Six crucial cultural elements have been pinpointed to boost the practice of speaking up behavior in interprofessional working and learning. Remarkably, hierarchy should not be held responsible as the wrongdoer; instead, can be a great facilitator through respect and appreciation. We propose that employing transformational and humble leadership styles can provide guidance on effectively integrating the identified cultural elements into the workplace and provide an IMOI framework for effective interprofessional speak-up beyond patient safety.

## Introduction

*Healthcare professionals are increasingly highlighting systemic shortcomings within the medical culture* (1, 2). *Current efforts to improve communication such as ‘speak-up’, often focus on techniques and isolated training programs within the patient safety context, whereas sustained improvement requires a culture shift* (1, 3, 4). *Attempts to foster such cultures require medical staff to change their habitual patterns, which in practice is a difficult task, even when speaking up is explicitly encouraged* (5, 6). *In order to create a sustainable set of consistent practices for interprofessional working and learning, it is necessary to understand the underlying cultural elements that influence speak-up beyond the patient safety context*.

In literature, speak-up usually refers to healthcare professionals raising concerns to draw attention to behavior or actions that pose a genuine risk to patient safety (7). The existing speak-up literature already provides valuable insights on the personal factors that can hinder or promote speak-up in the patient safety context (8). When healthcare professionals believe that speaking up will result in meaningful change to patient safety, they are more likely to do so (9). Conversely, they are less likely to speak up when patient safety is not at risk, even if not speaking up would come at the expense of personal (e.g., stress) or organizational interests, such as hindered interprofessional working and learning (6, 8, 10–12). Previous research has identified organizational culture, personality traits, and their interactions as important factors influencing the likelihood of speaking up within the patient safety context (6, 8, 10, 13). For example, a culture that values openness and encourages feedback is more likely to promote speak-up, while personality traits such as introversion may make individuals less likely to speak up in certain situations (14). Although there has been considerable research on speak-up and its benefits, the specific cultural elements important for speak-up beyond the patient safety scope within the context of interprofessional working and learning has received less attention.

Promoting a culture of open communication within interprofessional healthcare teams is of paramount importance, given that (1) traditional practices and the isolated acquisition of knowledge and skills are no longer effective amidst the rising challenges of multi-morbidity and aging patients, and (2) there is a growing emphasis on improving the patient experience, ensuring the well-being of healthcare professionals, and optimizing the overall performance of the healthcare system (15–18). Healthcare providers must work collaboratively across various professions to thrive in this ever-evolving landscape, not only in terms of content (e.g., diverse protocols across various specialties) but also in terms of relational aspects, enabling speak-up, deliberation, and continuous improvement and learning.

Simulation training aimed at promoting speak-up have been effectively used to enhance patient safety (4, 11, 19). Furthermore, various global initiatives have been launched to foster speak-up in hospital departments (6, 20). However, these efforts are typically *ad-hoc* or one-off activities within the patient safety context or overlook the crucial role of organizational culture in the process (20). Implementation research has shown that such activities are seldom effective in the long term and often fail (21). Taking a cultural perspective could potentially result in sustained improvements of these challenges, particularly as numerous healthcare scandals have surfaced as significant drivers for instigating cultural transformation. This is exemplified by the NHS’s initiation of the “Freedom to speak up” campaign in 2015, which was prompted by such scandals (22).

Although the available speak-up tools have been proven useful, e.g., for training purposes on the short term in the context of patient safety, healthcare organizations would still benefit from a durable approach to open communication beyond patient safety.

Understanding how cultural factors influence open communication, especially within a broader interprofessional context, can provide valuable insights to bridge existing gaps. This knowledge is crucial for fostering a potential cultural shift that optimizes learning. Therefore, our research question is: What is the impact of cultural elements on the ideal speak-up behavior as perceived by interprofessional teams beyond the patient safety context?

## Methods

### Study setting and design

The research took place in a general hospital in the Netherlands and focused on the Obstetrics and Gynecology department. This department was selected because interprofessional teamwork is inherent in this field due to the nature and high risks of the job, involving midwives, gynecologists, residents, and nurses etc., and, as a result, well-suited for investigating our research question (17). To gather information, an exploratory qualitative interview study design was used. This approach enabled the researchers to gain a comprehensive understanding of participants’ beliefs and thought processes that facilitated or impeded their ability to implement speak-up effectively (23).

### Participants and procedure

We used purposeful sampling to select the stakeholders. The participants were selected by two residents who were part of a speak-up workgroup in hopes of generating appropriate and useful data. Two or three stakeholders of each relevant stakeholder group were selected to form a representative sample of the team in the department.

**Figure 1:**
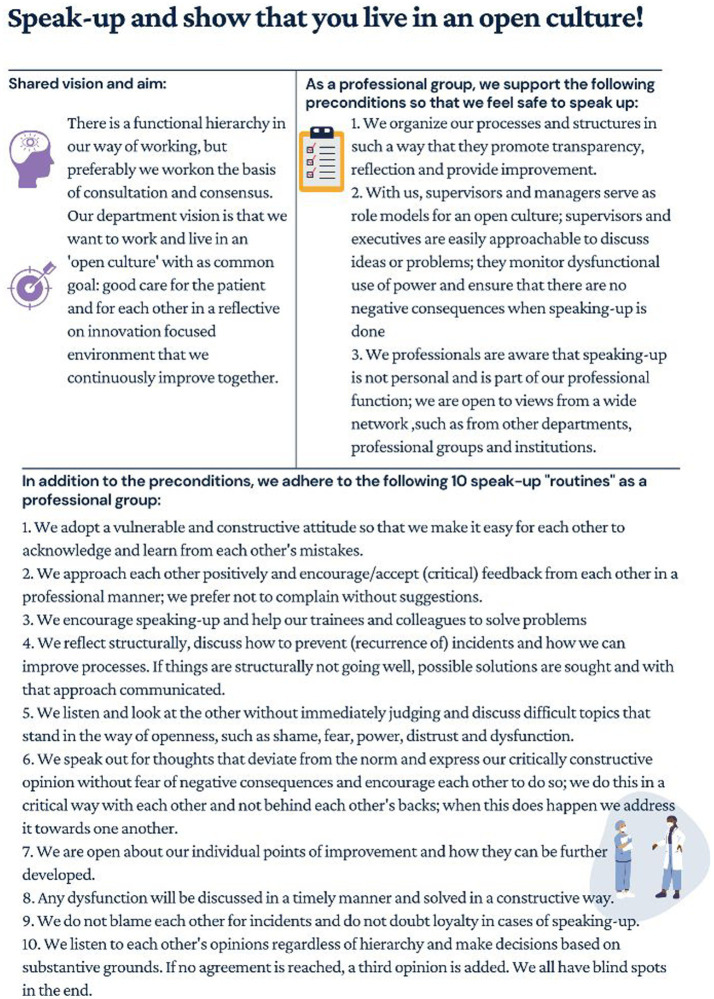
Speak-up pledge.

In order to investigate the impact of cultural elements on speaking up, it was crucial to establish a clear objective and ensure a shared understanding of the ideal concept of speak-up. Therefore, the research team developed a “speak-up pledge” (Figure 1) to prime participants and facilitate the interview discussions. This pledge was developed using speak-up literature and was refined through iterative discussions with the research team. It outlines ‘the way we communicate things around here’, including a shared vision, clear objectives, preconditions, and general routines for open communication.

From June to August 2020, the research team conducted telephonic semi-structured one-on-one, in-depth interviews with various stakeholders, including nurses, midwives, medical specialists, residents, and operational managers of the department. The operational managers were considered as members of the team for their role as coordinating foremen. All participants were women. After identifying potential participants, fourteen were invited to participate via email, which included attachments providing information about the study, ethical considerations, the speak-up pledge, and the informed consent form. The interviews were scheduled at times convenient for the healthcare professionals, with a clear timeframe established to ensure the interviews could be completed within the scope of the research. After conducting 13 interviews, data saturation was achieved within the initial cohort, with saturation evaluated by determining the amount of new data generated by each transcript (23). Therefore, the fourteenth interview was not carried out. To prevent misunderstandings, interviewees were provided with a summary of the interview within one week of its completion for approval. Participants were informed about their right to withdraw from the study at any time, and provided written consent indicating their understanding of the study’s aims and their voluntary participation, as well as for audio-recording the interview and publishing of data.

### Data collection

The interviews were guided by a topic list, following and derived from implementation and organizational culture literature. These concepts combined with concepts on social cognitive and behavioral theory were the units of analysis (Supplementary Material S1). Questions were asked about the current organizational facets to examine whether change is somehow required to successfully act according to the pledge, or a situation wherein healthcare professionals do no longer experience difficulties to speak up. Most questions during the interviews were built around the core question: what do people need from others, from the organization, from and for themselves to be ready for change and to act upon the speak-up pledge. Gaining insight into people’s current speak-up behavior, their strengths and weaknesses and what they consider most decisive for a successful implementation was considered necessary information to answer the research question. Other questions were related to a general view of the department, in order to comprehend the contextual factors of speak-up behavior and to make conversation (e.g., built rapport). All interviews lasted for approximately 30 to 60 min.

### Data analysis

Each interview was transcribed verbatim, and then analyzed using a three-step coding approach. The first step involved descriptive coding by reading through the data and identifying recurring concepts in at least three interview transcripts. Codes were created based on meaningful text, parts, or statements. Subsequent axial coding led to the creation of theoretical categories. Finally, selective coding was applied to aggregate the theoretical categories, resulting in the development of six themes.

## Results

Thirteen interviews were conducted with a range of interprofessional team members including nurses (*n* = 3), gynecologists (*n* = 2), midwives (*n* = 3), residents (*n* = 3) and (operational) managers (*n* = 2). Six themes were identified. The first theme highlights the importance of ensuring a shared vision on speak-up among interprofessional team members. The next three themes focus on three underlying cultural elements: appreciation, cohesion, and open-mindedness, which were identified as crucial competencies for successful speak-up behavior. The last topic explores practical considerations that may contribute to a culture shift by embedding cultural factors alongside conversational techniques in interprofessional working and learning. In the following sections, we will provide further detail on these themes.

### Managing vision

Although the speak-up pledge was utilized as a tool during the interviews, it is noteworthy that respondents commonly resonated with the content and acknowledged the inherent cultural significance associated with the pledge. They regarded it as a valuable instrument for delineating vision and expectations surrounding speak-up and fostering deliberate communication. It was also viewed as a useful resource for educating new colleagues on communication systems within the hospital. However, some respondents acknowledged that not everyone may adhere to the pledge. To ensure professionals can effectively speak up, a shared vision appealing to the diverse professional groups and support from supervisors and leadership were identified as crucial factors. In particular, these respondents emphasized the importance of leadership modelling and promoting the pledge to encourage its adoption.

“And for each professional group or department, there is a necessity to create a sort of an umbrella vision. Because we have nurses, midwives, residents, residents not in training, the gynecologists … everyone has their own thing and that's nice and okay, but how are we going to manage that… I miss that, little attention is paid to it in my experience.” (Midwife)

### (Dys)functional hierarchy

The functionality of hierarchy in facilitating speak-up stems from the diverse knowledge, experience, and effective decision-making it encompasses. Respondents generally acknowledged that hierarchy can be functional as long as the distinct roles within interprofessional teams are respected. In such cases, hierarchy served as validation for speak-up, contingent upon mutual respect. Respondents emphasized the importance of feeling appreciated and heard during decision-making processes, both within interprofessional teams and among teams within the same profession. However, hierarchy can also become dysfunctional when individual perspectives are devalued or ignored. A significant barrier to speak up was reported by the majority of respondents, who had experienced the detrimental effects of power dynamics and dependency within hierarchical structures.

“I do not believe that there should be a flat organizational structure. I think that's a bit nonsense actually. I don't think someone who is about to retire who is been in gynecology for 40 years is equal to me. I absolutely don't think so. They have a lot more knowledge and a lot more experience. So I think they should also be able to stand higher than me, for me to look up to. However, I don't think that should stop you from having a discussion with that person.” (Resident)

Therefore, leaders need to actively participate in work processes to ensure that they are easily approachable for discussion and need to be mindful of the influence their positions of power have on the work floor, by exhibiting appropriate behavior, vulnerability, and careful language towards their colleagues.

### Robust interpersonal relationships

According to respondents, the quality of relationships between professionals affects their willingness to speak up. When professionals work together for an extended period or engage in interprofessional team activities, they gain insight into each other’s strengths and weaknesses, which can encourage speaking up.

“I myself believe that you have to know each other for speaking up. I dare to speak up against all gynecologists. (…) Also just that they know me by name too, that they really see me that they are just, you know, straight up. That honestly makes me feel like I’m treated fairly.” (Nurse)

Conversely, lack of familiarity between colleagues can hinder speak-up, particularly for less experienced professionals who may doubt the acceptance of their input.

Young professionals in learning positions are particularly vulnerable due to their reliance on grades and future career prospects. However, respondents also noted that residents tend to develop a more positive attitude towards nurses as they gain experience working with them, regardless of age or experience. Some respondents even suggested that younger professionals could influence the culture positively by speaking up, despite resistance from older colleagues who are less likely to change.

Furthermore, the nature of the relationship and the context in which speak-up occurs are also important factors that influence speak-up behavior, in addition to the duration of the relationship. Informal contact outside of work is considered to be a stimulating factor in increasing cohesion among healthcare professionals. One stakeholder noted that current forms of informal contact are limited to peer-to-peer interactions or within the same profession, and suggested that more interprofessional gatherings should be organized. For example, the residents and gynecologists engage in joint activities like skiing, without involving the nurses. But even the lack of interprofessional lunches, where gynecologists for example could have meals with nurses, is a minor yet noteworthy factor.

“You know when it’s safe, in an honest and safe family, you can share your distress and say ‘I think you’re doing it wrong’ or something like that. But, it really needs to be safe and honest and that’s not quite the case. And the logistics, like I said. You should have much more consultations and meeting moments. You should really know each other.” (Nurse)

### Strategic speak-up strategy

Most respondents believe that speaking up about patient-related matters or medical discussions is less problematic than addressing non-patient-related behavior. However, many respondents choose not to speak up about non-patient-related behavior to maintain a good or at least not worsen an already less satisfactory relationship. Although speaking up is considered professional, it is often taken personally in such contexts, and it can impact the relationship negatively in the long run.

“… you know, when you’re dealing with a person with whom you don’t share a nice relationship or merely a good work relationship, then I would really watch my words carefully. And yes, that isn’t really safe of course; yet it happens a lot, I would say more than half of the time.” (Midwife)

Healthcare professionals strategically choose when to speak up and may choose to avoid certain situations to maintain relationships. This is especially true for situations outside of patient care where feedback may be taken personally and negatively impact the relationship. Additionally, the level of liking or disliking someone influences the ease of giving feedback. Healthcare professionals are more likely to speak up to someone they like as feedback is perceived to be given with good intentions, whereas they may avoid giving feedback to someone they dislike or do not have a good rapport with. This dynamic can be present in both hierarchical and peer-to-peer relationships, as well as relationships between departments.

“Well, that you feel safe to express things and not afraid of being punished or that you get hit on the head. I do not feel fear with the doctors, but I do have some fellow nurses where I do not dare to say anything because they just have very strong opinions. (…) otherwise you will have to take the bullet and you just don’t feel like doing so.” (Nurse)

Respondents also noted that not speaking up and being silent on a topic can translate in gossiping. While gossiping or speaking negatively about others behind their backs is often considered a natural human tendency that can provide a sense of relief by releasing pent-up emotions or feelings, it can also lead to the formation of biases and prejudices.

“We regularly talk critically about each other behind each other's backs. (…) We just like gossiping too much. Listening could sometimes really be better before immediately judging. (.) Not everyone speaks as smooth or fast as others so that should not be the bar. I think everyone should be aware that not everyone is alike; everyone I work with has unique qualities, you cannot say everyone should be like me.” (Resident)

### Open-mindedness as a professional attitude

The majority of respondents tend to view themselves as approachable for feedback while perceiving others as unapproachable.

“It depends on how people deal with it, if I give feedback, not necessarily positive feedback, I notice there is tension, because the person who has to receive it must also be able to hear it and be able to do something with it and take it seriously (…) and it should not be that you cut off someone’s head or have a judgment. So it is really about honesty and the way of saying things and how you deal with it as person to person.” (Midwife)

Overall, two important messages were emphasized: the importance of open-mindedness and psychological safety. Open-mindedness was described as the ability to listen without judgment, while psychological safety was described as feeling safe to speak up without fear of punishment or judgment.

When individuals possess open-mindedness, it can foster a sense of psychological safety among them. The ability to feel psychologically safe is determined by multiple factors that relate to open-mindedness, such as the ability of the feedback giver to communicate the message in an honest and skillful manner, the feedback giver’s expectations of how the receiver will process the feedback, and how the receiver ultimately responds to it. Open-mindedness allow team members to be transparent and vulnerable by fostering trust, respect, and adaptability. Ultimately, this enables team members to feel comfortable enough to express their thoughts and feelings openly. Reflecting together and sharing thoughts can foster mutual understanding, but respondents suggest that interprofessional reflection is infrequent. Furthermore, treating speak-up as a professional competence instead of a personal message to an individual may make it easier for people to engage in it or identify when others are doing it.

“I don't care who you are as a person, it shouldn’t matter the other way around, we really need each other to work together so it's just really so important not to treat each other like that. You work as a professional (…) you shouldn't let those emotions you have about each other get in the way.” (Resident)

Moreover, it may also be exacerbated by certain group dynamics. For instance, one respondent points out that despite the evolving dynamics and increasing female emancipation, men continue to hold a dominant position over women in discussions and the final decision-making process, while another respondent mentions that a group comprised mostly of women may be susceptible to specific female characteristics that affect the way they work together.

“It's a different group, a different dynamic, maybe because it's mostly women, I don't know but everyone has to have an opinion on everything, it takes a lot of time for them to agree with each other.” (Organizational Manager)

### From training to practice: cultural practices to embed in interprofessional working and learning

While there is a willingness to change and reflect, concrete action towards encouraging speak-up behavior is still lacking. Although the hospital desires an open culture, employees are not provided with the necessary tools to achieve this.

Respondents report that open communication is only emphasized during acute situation training in the patient context, which occurs once or twice a year, with no other initiatives to stimulate open communication and a lack of tools on how to successfully speak up. In this training context, participants usually speak up without hesitation as it serves the aim of the training and there are no personal stakes involved, even in working environments where speak-up is typically discouraged due to power imbalances. However, outside of this particular context, the level of perceived difficulty and emotions as well as the absence of relevant professional skills on both the part of the person speaking up and the person receiving the message influences the decision to speak up or remain silent.

“Because you lack those skills to raise your concern properly, so then you often decide to just let it go.” (Resident)

To bring about effective open communication, it is important to provide tools that facilitate the development of specific competencies, including conversation techniques on how to speak up, non-judgmental listening, coping with emotions, and interprofessional reflection on practice.

“There are feedback courses for the trainers and residences, however, interprofessional feedback courses lack (…) For example, a nurse has never learned what feedback they can give to a gynecologist. (.) They are not sufficiently supported and trained in it.” (Medical Specialist 2)

The physical context in which healthcare professionals work seems to have a significant impact on their experiences, both formally and informally. One notable contrast is between two locations, where interprofessional teams work together in one room on one location, and nurses and doctors work separately in different rooms on the other. The former setting is generally viewed as advantageous for relationships and communication channels.

“There should be more mutual understanding here. And I really think that this is something that is reinforced by the way the logistics are arranged, in that the nurses are in one room and the doctors in a separate one. It really feeds the sense of disunity”. (Resident)

## Discussion

This study explores the impact of cultural factors on speak-up behavior in interprofessional teams beyond patient safety. The study identified six themes that enhance speak-up behavior: the importance of vision management, functional hierarchy, interpersonal relationships, healthcare professionals’ speak-up strategy, open-mindedness as a professional attitude, and cultural practices to embed in interprofessional working and learning. These themes can be categorized into four levels; the professional, interprofessional, leader, and organizational levels. Firstly at the professional level an open-minded professional attitude was considered vital for effective communication, next to a corresponding strategy showcasing speak-up on specific subjects while exercising restraint on others. At the interprofessional level, robust relationships between individuals played a crucial role in encouraging speak-up behavior, particularly in situations where power differentials exist. At the leader level the majority of respondents recognized that hierarchy proves effective when professional opinions are sufficiently respected and valued. Finally at the organizational level, having a shared vision that encourages speak-up is beneficial for alignment, and embedding specific cultural elements into interprofessional working and learning can positively influence speak-up behavior across all levels.

The study’s findings are intriguing as it provides new perspectives on existing knowledge regarding team effectiveness in the context of interprofessional speak-up behavior beyond patient safety. Theorists have extensively discussed what makes some teams more effective than others, initially focusing on the outcomes of team performance and viability (24). Eventually, attention shifted to processes that explain why certain inputs affect team effectiveness and viability. However, a single-cycle linear model no longer suffices, as effective teams now operate more than ever within complex, fast-changing, multilevel systems across various times, tasks, and contexts. Ilgen et al. (24) for example, expanded the literature with their widely used Input-Mediator-Output–Input (IMOI) model of effective teams, proposing a broader scope of mediating processes. They also introduced the concept of a cyclical causal feedback loop, indicating that outcomes influence and may serve as initial inputs. Each team experience impacts the next, as both the team and its members grow during these processes. The current landscape of teambuilding is evolving towards greater emphasis on interprofessional collaborative efforts aimed at improving healthcare outcomes and reducing costs. Emerging concepts like shared care, shared leadership, and shared decision-making over the past decade demonstrate this shift (25–27). As a result, interprofessional practices are expanding beyond traditional teams, even though their effects are not conclusively proven (28).

This makes the way professionals interact with each other in the various organizational layers, as well as between different professions, departments, and even different organizations, particularly important, where, according to our findings, hierarchy does not have to be inherently problematic, contrary to previous research (4, 5, 10, 13, 17, 29, 30). The participants in this study acknowledged the functionality of hierarchy when it comes to decision-making for example. Nevertheless, the study emphasizes that the underlying problem lies in the core values and norms surrounding behavior within the hierarchical structure; even with the establishment of a collective vision, there is a challenge in fostering a sense of value for individuals’ opinions. These findings align with research by Vatn et al. and Weiss et al. in the healthcare sector (31, 32), but is also consistent with literature in organizational science (33). Bunderson and Reagans found that while power and status can complicate collective learning by disrupting shared goals, risk-taking, and knowledge sharing, the socialized use of power can actually leverage social hierarchy to enhance collective learning (33), making it an essential requirement for leaders in environments where learning is key and power and status differences exist. For leaders to become skilled in the socialized use of power, they must embrace leadership styles that extend beyond traditional theories (34). Considering our results and the literature, hospitals might benefit from concepts such as that of ‘Transformational Leadership’, ‘Humble Leadership’, and ‘Psychological Safety’, as described by Jacobs, Schein and Edmondson, respectively. These concepts are relevant to the study’s findings as they address the identified topics of a shared vision, cohesion, appreciation, open-mindedness and leadership approaches that appear necessary to confront the challenges of speak up behavior (35–37).

Transformational leadership is described as a form of leadership that inspires and motivates followers to achieve outcomes beyond expectations, helping followers to grow and develop by responding to their individual needs (38). Jacobs’ suggests managers to focus on changing the thought processes that drive behavior by establishing agreement on a set of guiding principles and believes that this will naturally lead to the desired actions from others (35). Humble Leadership emphasizes the development of personal and cooperative relationships, openness, and trust, as opposed to the traditional approach maintaining an appropriate professional distance (37). The focus is on interpersonal and group dynamics, where leaders embrace ambiguity and work to reduce the distance between opposing sides to establish shared commitment based on openness and trust. Simultaneously organizational leaders can enhance their awareness of on-the-ground happenings by cultivating and sustaining effective exchange relationships with frontline employees (39). When properly employed and supported by leaders, this approach is thought to help others feel valued despite the hierarchical structure and improve relationships by actively managing them, encouraging open communication, and preventing dishonest feedback to save face, especially among peers (37). Schein recommends that all teams, regardless of size, perform better when their members feel psychologically safe to share their thoughts and ideas with one another. Psychological safety as to the description of Edmondson refers to an environment where individuals feel secure enough to take interpersonal risks by expressing their concerns, questions, or ideas without fear of negative repercussions (36). This includes being candid and willing to engage in constructive conflict to learn from various viewpoints, while also having the assurance that others will give them the benefit of the doubt when they admit to making a mistake or need help (36).

**Figure 2:**
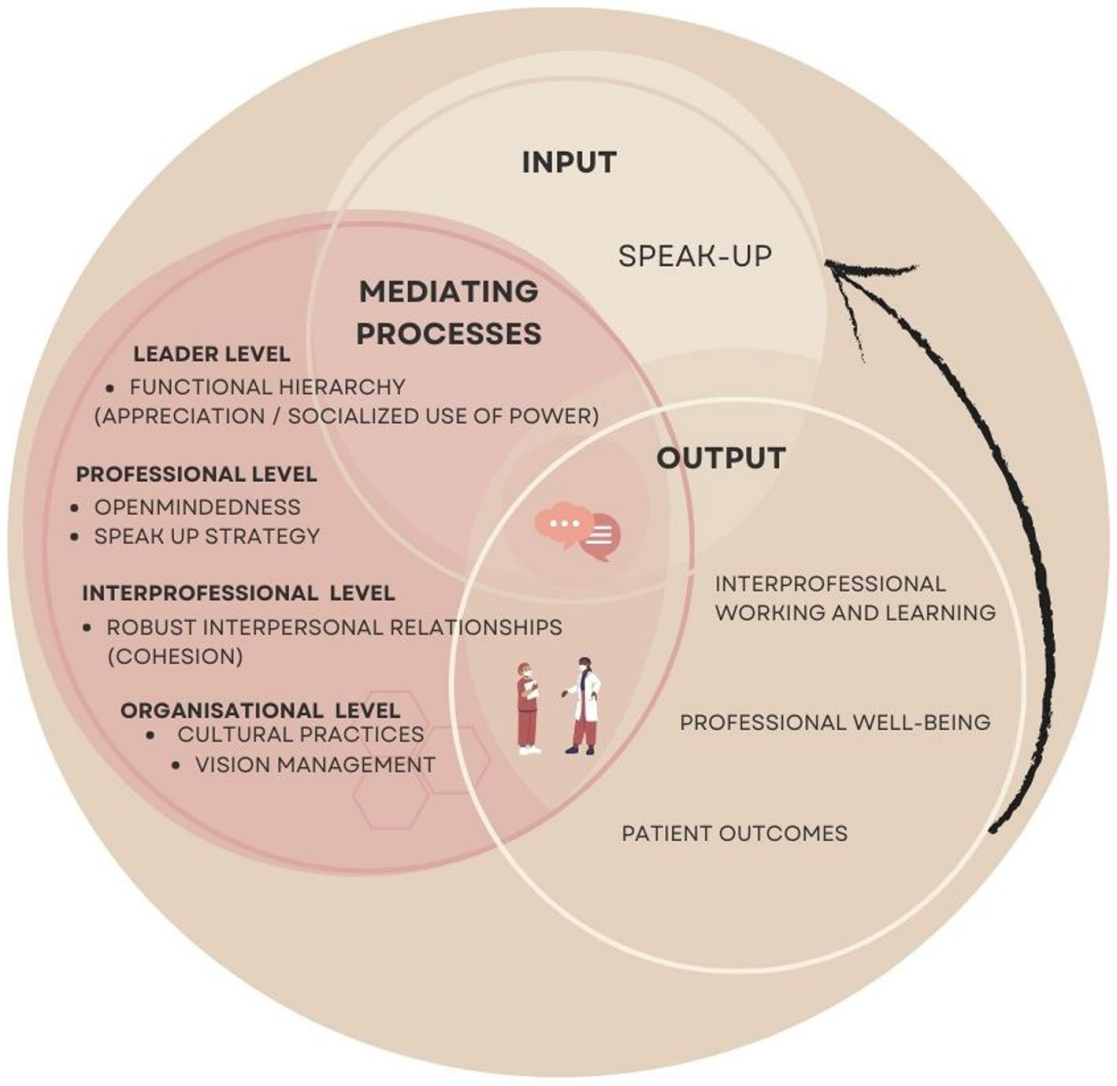
IMOI framework for effective interprofessional speak-up beyond patient safety.

Whereas the concepts of transformational and humble leadership give emphasis to this study topics of shared vision, cohesion, respect, and openness, the concept of psychological safety relates to the cultural element open-mindedness, which can be seen as a professional attitude that is vital for success. Implementing the aforementioned concepts could facilitate hierarchical structures to become more conducive to effective interprofessional working and learning, yet remains a formidable undertaking in reality. Creating an environment of open communication and open-mindedness, free from prejudices and with a willingness to give others the benefit of the doubt, remains a challenging task. A recent scoping review has identified how team members internalize biases related to dominance and expertise, and how team members adapt to these biases in dynamic ways, resulting in negative effects on interprofessional collaboration (40). In Figure 2, we present an IMOI framework designed to facilitate effective interprofessional speak-up beyond patient safety, emphasizing the need for various mediating processes to enhance interprofessional working and learning, professional well-being, and patient outcomes. The resulting positive outcomes can, in turn, foster a speak-up culture, encouraging further input and engagement.

## Recommendations

*Knowledge—*Traditional interprofessional training often focuses on general attitudes towards working together and the acquisition and demonstration of knowledge and skills (18). To promote effective speak-up behaviors within interprofessional teams, it is important to equip them with technical tools that are tailored to their specific needs. One such tool is the “Humble Inquiry” approach, which emphasizes the use of curious and open-minded questioning (41). This approach requires the questioner to be vulnerable and willing to learn from the responses of others and may be used for speak-up in a subtle way. Ongoing training and education on this topic, in which the six cultural themes found in this study are emphasized, should be key in facilitating interprofessional working and learning. Examining the effect on interprofessional practice is a subject for future research.

*Reflective practice*—Interprofessional teams must learn to minimize biases, acknowledge and appreciate the valuable insights and perspectives of all members. Interprofessional working and learning that incorporates a shared understanding of roles and responsibilities may contribute to this (42). Other studies have identified that encouraging reflexivity, or reflecting on one’s own professional identity and biases, can improve interprofessional working and learning (43, 44), by making people unconsciously suppress their own perspective for that of another (35). Having such a mind-set not only prepares the team for speaking up, but also for receiving feedback and engaging in reflective practices together (45). The socialized use of power is a fundamental prerequisite in this context, particularly because power and status differences can influence the willingness of members to participate in collective learning activities. These differences can impact their perceptions and feelings of psychological safety, their risk assessments, and their inclination to take initiative and independent action (33).

*Consolidation in cultural patterns –* The incorporation of the six themes uncovered in this study has the potential to be considered as targets for future interventions and bring about a desired shift in culture, promoting speaking-up, and interprofessional working and learning. Appelbaum et al. (18) arrived at a parallel finding indicating that psychological safety and power distance can serve as substantial factors driving cohesion and fostering effective collaboration. However, organizations often fail to address the motivational and supportive factors that impact the target group when preparing for change. As a result, implementation efforts are often unsuccessful in bringing about the desired behavioral change (21). Achieving effective interprofessional working and learning is only possible with the provision of optimal organizational support and resources. This requires individual transformation as well as changes in collective management and leadership practices. Therefore, leaders must possess the ability to envision the organization’s role and lead transformational efforts that align with the purpose of embedding the cultural aspects. So first of all it is crucial to establish a shared vision, such as through a speak-up pledge, and ensure that the organization has the ability to effectively receive and respond to feedback (37). To foster effective communication and relationships, leaders can be trained using transformational and humble leadership styles. But most importantly, they play a crucial role in establishing habitual patterns that drive a cultural shift.

## Strengths and limitations

The aim of this research was to provide valuable insights into the cultural influences on speak-up behavior. One of the strengths of this study lies in its comprehensive examination of the potential obstacles associated with implementing speak-up behavior, all the while recognizing the paramount significance of cultural factors in this context.

A limitation of this study is that it was conducted in only one hospital in one country. Although we believe in some transferability of the findings in similar healthcare systems and departments, the complexity of organizational culture means that findings may not be fully transferable to other countries. Therefore, it would be valuable to explore underlying cultural factors in different settings, not just in healthcare but also in other industries. Furthermore, a limitation lies in the selection of respondents, as the two residents who selected the respondents could potentially introduce bias. Respondents’ participation and responses may also be influenced by this approach, thus contributing to potential bias. To mitigate this bias, we sought to minimize it by having interviews conducted by an external individual who is not affiliated with the healthcare profession.

## Conclusion

This study explores the impact of cultural factors on speak-up behavior for interprofessional working and learning. Six key cultural elements have been identified as enhancers of speak-up behavior within interprofessional working and learning. Rather than assigning blame, hierarchy can serve as a valuable facilitator. Emphasizing managerial vision, in addition to vital traits such as open-mindedness, is imperative. The incorporation of the themes uncovered in this study has the potential to bring about a desired shift in culture, promoting speaking up, and interprofessional working and learning. Transformational and humble leadership approaches offer valuable direction for applying this knowledge in the workplace and strategically utilizing the act of speaking up.

## Data Availability

The raw data supporting the conclusions of this article will be made available by the authors, without undue reservation.
