# Supplementary material for: How do cultural elements shape speak-up behavior beyond the patient safety context? An interprofessional perspective in an obstetrics and gynecology department
Source: Front Med (Lausanne). 2024 Sep 4;11:1345316. doi: 10.3389/fmed.2024.1345316 (PMC11409420; doi:10.3389/fmed.2024.1345316)
Supplement: Supplementary file 1 [file Data_Sheet_1.pdf]

SUPPLEMENTARY MATERIAL S1. TOPIC LIST & INTERVIEW GUIDE

TOPIC LIST

Transformational concept related topics (except for first topic)

Influence of the external environment on speak-up behaviour
Influence of the hospital's mission and strategy on speak-up behaviour
Influence of leadership on speak-up behaviour
Influence of organizational culture on speak-up behaviour

Transactional concept related topics

Influence of the hospital's procedures and rules on speak-up behaviour
influence of management practices on speak-up behaviour
influence of the hospital's organizational structure on speak-up behaviour

Personal and interpersonal related topics (except for last topic)

Influence of the working climate on speak-up behaviour
Influence of the behavioural capability on speak-up behaviour
Influence of values and needs on speak-up behaviour
Influence of the motivational level on speak-up behaviour
Influence of the individual and general performance outcomes on speak-up behaviour

INTERVIEW GUIDE

Topic 1: Speak-Up Behaviour

How would you describe your own speak-up behaviour?
How could you improve your speak-up behaviour?
What is your first impression of the speak-up pledge?
What elements do you find more appealing than others? Why?

Topic 2: Context

External environment

Aspects that could influence your readiness to act for change in context of the speak-up pledge.
What external influences do you consider important enough to influence your readiness to bring the speak-up pledge into practise? Why?
What do you further need from the external environment to stimulate you acting upon the pledge?

Outcomes

What outcomes would motivate you to change your behaviour to the pledge? Why?
What outcomes are less important for you as individual? Why?

Mission and strategy

What is according to you the mission and strategy of the hospital?
How does speak-up align with the vision and strategy of the hospital?

Organizational culture

How would you describe the organizational culture of this hospital?
How does the organizational culture influence your readiness to speak up according to the pledge?
When we still talk about culture, what would you like to be different, in order for you to be ready for implementation? How could this be realized?

Rules and Procedures

How do the rules and procedures contribute to you being more willing to engage in speaking up behaviour? How?
What change would be needed concerning the rules and procedures to stimulate your readiness for change? Why?

Organizational structure

How would you describe the organizational structure of this hospital?
Would you as consequence of the current structure be ready to implement speak-up according to the pledge into practise? Why (not)?
How would you describe the current communication channels?
How do the current communication channels align with the speak-up pledge? How would you like to improve this?
What would be your role in this?

Working climate

How do you think your team members or colleagues perceive speak-up behaviour? Why?
What would your colleagues think of implementing the speak-up pledge?
What would you need from them for a successful implementation?

What do you think they would need from you?
How would you like to meet those needs?
Also, how ready are you, skills-wise, to implement speak-up according to the pledge?
What would you further need to be capable of implementing speak-up according to the pledge successfully?
Who would play a role in that process?

<u>Topic 3: Leadership</u>
How would you describe the leadership style in the hospital?
How would you describe the role the leaders have to stimulate your readiness for change?
What would you need from your leaders to successfully bring speak-up according to the pledge into practise?
How is the leadership team currently equipped to do that?
Would you be ready to implement speak-up according to the pledge with the current attitude managers have towards speaking up behaviour? Why (not)?
What would you need from your manager to change that?/ to improve, in order for you to act upon speak-up according to the pledge?

<u>Topic 4 Personal and interpersonal related topics / values and motivational level</u>
What would still deprive you from implementing speak-up according to the pledge, if all aspects were covered? Why?
What would drive you to implement speak-up according to the pledge? Why?
We talked about hospital outcomes, but what effect do you think the implementation of pledge will have on your own (quality of) work?
How important do you consider these effects for putting speak-up according to the pledge into practise?
What possible effects are worrying you?
Is there anything else that is worrying you while thinking of implementing the pledge? Why?

Final questions:

What factors do you consider most decisive in determining a successful implementation for the speak-up pledge? And why do you think that?
